# The Negative Impact of Noise on Adolescents’ Executive Function: An Online Study in the Context of Home-Learning During a Pandemic

**DOI:** 10.3389/fpsyg.2021.715301

**Published:** 2021-09-22

**Authors:** Brittney Chere, Natasha Kirkham

**Affiliations:** Centre for Brain and Cognitive Development, Department of Psychological Sciences, Birkbeck, University of London, London, United Kingdom

**Keywords:** environmental noise, home-learning, adolescent development, COVID-19, online research, executive function

## Abstract

UNICEF estimates that 1.6 billion children across the world have had their education impacted by COVID-19 and have attempted to continue their learning at home. With ample evidence showing a negative impact of noise on academic achievement within schools, the current pre-registered study set out to determine what aspects of the home environment might be affecting these students. Adolescents aged 11–18 took part online, with 129 adolescents included after passing a headphone screening task. They filled out a sociodemographic questionnaire, followed by a home environment and noise questionnaire. Participants then completed three executive function tasks (the Flanker, the Backward Digit Span, and the Wisconsin Card Sorting Test) while listening to a soundtrack of either white noise or home-like environmental noise. For purposes of analysis, based on the noise questionnaire, participants were separated into quieter and noisier homes. Results revealed that measures of the home environment significantly correlated with individual perceptions of noise and task performance. In particular, adolescents coming from noisier homes were more likely to report that they studied in a noisy room and that they were annoyed by noise when studying. In terms of noise and task performance, the Flanker task revealed that while older adolescents were more efficient overall than their younger peers, those older adolescents from noisier homes seemed to lose this advantage. Additionally, reaction times for younger adolescents from noisier homes were less impacted by accuracy compared to their peers from quieter homes, though there was no difference for the older adolescents. This evidence suggests that higher in-home noise levels lead to higher rates of annoyance and may be hindering home-learning, with both younger and older adolescents being impacted. Furthermore, the long-term effect of in-home noise on adolescent executive function task performance indicates that these findings transcend the pandemic and would influence in-school learning. Limitations and advantages of online adolescent research without researcher supervision are discussed, including sociodemographics and adapting tasks.

## Introduction

Due to the COVID-19 pandemic, schools across 188 countries closed their doors to students by April 2020 in order to contain the spread of the virus, leaving approximately 1.6 billion students to continue their education from the safety of their homes^1^. The impact this will have on the education of these children is vast and unprecedented. One particular question that needs to be addressed is how the change in environment, going from the structured classroom to the home, may be affecting educational outcomes. Secondary schools are often purpose built to foster learning, from the design and functionality of the entire building to the individual sections within classrooms (for an overview of U.K. regulations, see Department for Education and Skills, 2015). Importantly, the infiltration of noise from the outdoors and the transmission of noise between rooms within the building is often largely reduced. Even then, however, there is a large body of evidence showing that students’ ability to learn is negatively affected by imposing noise, and that there may be possible long-term cognitive consequences (for an overview see Shield and Dockrell, 2003; Klatte et al., 2013). What, then, could this mean for learning within the home, an environment that is built to serve various functions and with the potential of having many and different distracting noise sources?

While noise pollution is presently government regulated, many researchers in the field would argue that stricter regulations need to be implemented and that more research is necessary based on the documented adverse effects from exposure to noise (Fink, 2017). Currently, the U.S. Environmental Protection Agency’s general population guidelines are that the maximum average exposure to noise should not exceed 70 dB in order to prevent hearing loss, and that average indoor noise levels of 45 dB or greater will begin to interfere with activities and create annoyance^2^. In a study attempting to determine the exposure to noise in schools, recordings across 13 U.K. schools during lessons revealed that the overall average noise level was found to be 64.2 dB L_Aeq_, with a general background noise level of 51 dB L_A90_ (Shield et al., 2015). A study similarly attempting to establish the in-home noise levels of school children found that the average noise level in the main room of the home was 55.2 dB L_Aeq_ and the child’s bedroom was 48.2 dB L_Aeq_ (Pujol et al., 2014). Evidently, similarly to schools, home noise levels seem to be exceeding the recommended indoor noise levels, making adolescents at risk for noise-induced annoyance and hindered learning.

As most formal learning occurs within the school environment, much of the research on how environmental noise impacts on learning takes place within schools. Not until the pandemic has the home been the environmental base for formal learning, with hardly any previous research, to our knowledge, having looked at the effect of in-home noise on learning. Thus, we will review the research focused on adolescent learning within schools, and link this to the few studies that have measured general in-home noise levels to determine how these environments relate. The two streams of focus within the school literature are commonly the impact of noise on academic outcomes and the impact of noise on annoyance, with annoyance being defined as an emotional and cognitive response to a noise exposure (Guski et al., 2017) and is often used as an indicator for individual sensitivity to noise (Enmarker and Boman, 2004; Connolly et al., 2013). In a recent study by Massonnié et al. (2020) looking at the effect of noise on reported annoyance and schoolwork interference in children, they concluded that these are separate but correlated mechanisms that may be susceptible to individual differences. This last note concurs with Passchier-Vermeer and Passchier (2000), who determined that individual differences are key to understanding how noise affects development, particularly societal factors such as the home environment. Thus, while attempting to understand how in-home noise may be impacting learning using the research previously done within the school environment, we may further understand how adolescents’ individual experiences within their home environments might be impacting their school learning, allowing for both streams of research to inform each other.

Unfortunately, most of the literature on noise annoyance and its effects on development are focused specifically on road traffic noise (Massonnié et al., 2020), giving a very narrow understanding of noise-induced annoyance as it is a subjective measure that can vary individually depending on the type of noise (Enmarker and Boman, 2004). What is known, is that levels of reported noise have been directly tied to noise-induced annoyance, with higher levels of noise relating to higher rates of annoyance in adolescents aged 13–15 (Ali, 2013) and 11–18 (Minichilli et al., 2018), though another study with 13-to-15-year-olds only found a poor correlation (Lundquist et al., 2000). Furthermore, adolescents have reported annoyance to both external and internal noises (Ali, 2013), though interestingly, one study found that adolescents aged 11–16 reported more annoyance for noise stemming from outside of the classroom compared to internal noise, even though noise from within the classroom occurred much more frequently (Connolly et al., 2013). The positive deduction made by the authors was that reducing the nuisance of outdoor noise heard within the classroom alone should then greatly decrease the negative effects of noise. Potential evidence for this was found by Ali (2013), who reported that noise levels within classrooms significantly reduced after restricting nearby outdoor road traffic and railways.

The findings looking at effects of age on rates of noise-induced annoyance are not as clear, with some studies showing that younger adolescents report more annoyance compared to their older peers (Lundquist et al., 2000; Ali, 2013; Minichilli et al., 2018), while another study found higher rates of noise-induced annoyance in older adolescents (Connolly et al., 2013). While effects of noise on academic performance within the literature of noise-induced annoyance is often not directly measured, students have reported the belief that environmental noise levels have negatively affected their academics (Ali, 2013; Connolly et al., 2013). To note, no effects of gender on rates of annoyance have been found (Lundquist et al., 2000; Enmarker and Boman, 2004; Minichilli et al., 2018). We can therefore conclude that levels of indoor classroom noise are directly tied to rates of annoyance and self-reported academic performance. Such a finding is important, as Pujol et al. (2012) reported that indoor noise levels in the home have been linked to dwelling type, with children’s bedroom noise levels being higher in more collective dwellings (i.e., being closer to and having more neighbors) and the main room in the home being higher in detached dwellings (i.e., no direct neighbors). Furthermore, they reported higher noise levels when more people were present in the home. This is particularly poignant in the context of COVID-19, whereby families may be grouping together in homes in order to support each other through the pandemic and the isolating lockdowns.

The line of research that has been directly measuring the effect of noise on academic achievement has largely focused on children (Connolly et al., 2019), with two in-depth reviews published. In the earlier review by Shield and Dockrell (2003), they determined that noise within the classroom appears to impact specifically on numeracy, reading, language, and speech, and also on overall academics. Furthermore, they deduced that noise is likely to have a greater impact when completing tasks that require higher processing demands, meaning that adolescents with more cognitively demanding schoolwork may be more affected by noise than the children included in the review. In Klatte et al.’s (2013) later review, they concluded that there is currently evidence for significant negative effects of environmental noise within the classroom on auditory tasks that involve the perception of speech and listening comprehension, as well as non-auditory tasks that involve reading, writing, and short-term memory. Of note, both reviews determined that due to the mixed findings in the literature, there is not enough evidence for a strong understanding and conclusion on how noise negatively impacts on academic learning. They do, however, state that chronic exposure to environmental noise is likely to impact on general cognitive development, meaning that exposure during childhood could have cascading effects on later adolescence and potentially adulthood. This would also imply that consistent exposure to noise in the home could also have widespread consequences.

A more recent study specifically looking at adolescents aged 11–16 found direct evidence of classroom noise affecting their reading ability. Connolly et al. (2019) had students listen to a naturalistic recording of non-verbal classroom noise through headphones at 50, 65, and 70 dB while completing a short reading task. By using an audio recording that depicts the actual environment that students typically learn in, along with a controlled learning paradigm, they were able to directly determine how this noise impacts on performance. Interestingly, they found that while older adolescents were generally better than their younger peers in the 50 dB condition, only the older adolescents’ performance was negatively affected in the louder 65 dB condition. When comparing the audio at 50 and 70 dB, participants of all ages attempted less questions and were less accurate in the 70 dB condition. It was suggested that the greater effect of the 65 dB noise condition on the older adolescents was tied to their enhanced focus on the task, and they were thus more disrupted by the noise, but then at the highest noise level it was loud enough to be cognitively distracting for both age groups. It is therefore evident from much of the research looking into how environmental noise impacts on learning, that the cognitive ability to deal with background noise plays a key role.

When environments are information rich with lots of stimulus input, learning requires the skills to attend to and process relevant and important information, while ignoring the irrelevant and distracting information (Stevens and Bavelier, 2012). As Parmentier (2014) concludes in a review, auditory distraction is defined as an auditory stimulus that violates what the cognitive system has predicted, therefore taking away attention from the task at hand and interfering with the current goal-directed behavior. Thus, the ability to selectively attend to the appropriate information and inhibit the distractors is necessary for learning to occur in most environments, giving executive function (EF) a fundamental role. Importantly, research shows that EF undergoes significant development throughout childhood and adolescence (Diamond, 2013). So while much of the research has historically focused on children, it is clear that adolescents are still very much susceptible to the negative effects of noise. Importantly, EF has also been directly linked to academic achievement across development (Jacob and Parkinson, 2015), particularly in math and reading (Best et al., 2011). More specifically, working memory, attentional flexibility, and inhibitory control have been found to be key EF components in this relationship (McClelland and Cameron, 2019). EF therefore serves two important roles within the current literature: (1) its developmental trajectory implies that differences within younger and older adolescents should be seen in terms of how noise impacts on learning, and (2) it is key for successful academic learning. The question remains though- how might all of this research translate to how noise within the home environment is impacting adolescent learning?

An important aspect to consider when making the comparison between the school and the home, is that adolescent formal learning usually involves teacher-guided group or independent learning, with other students of the same age generally working on the same tasks. These are factors that may indeed help keep focus and reduce the effects of noise and distraction. The environments at home, especially during the pandemic, most likely do not have any of these protective factors. Learning is now partially ‘live’ through an online format with stints of it being fully independent work without any teacher supervision (note: this will be dependent on the type of school and the school system). Additionally, very rarely would there be another person in the home working on the same task. Instead, with entire families being confined to the home during the pandemic, students learning at home could be surrounded by parents taking work calls, younger siblings playing, and grandparents making a cup of tea. Furthermore, the home is built to serve many functions other than studying and learning, ranging from cooking food, cleaning clothes, relaxing, playing, practicing hobbies, and being entertained. Thus, unlike a school, the home is not built to foster academic learning and to block out noisy distractors. So, while it is clear that noise has a negative impact on annoyance and academic achievement within schools, it is important to consider that the effect in the general home environment may be even greater. Of further importance is the finding that lower SES homes are likely to be exposed to higher levels of noise (Dale et al., 2015; Casey et al., 2017), and more chaotic homes have been associated with lower family income and less educated caregivers (Dumas et al., 2005). This would imply that some adolescents may be more burdened by the impact of noise on their home-learning.

The main purpose of the current study was (a) to investigate the effect of the home environment, and in particular in-home noise, on adolescent learning in order to better understand how students have been impacted by the pandemic, and (b) to expand the methodology of online developmental research. To address these two aims, we ran an online experimental study along with questionnaires, where adolescents were asked to complete several EF tasks during which they listened to either a naturalistic recording of a noisy home or white noise. The EF tasks were used as a proxy for academic learning for two reasons: (1) the chosen tasks measure shifting, inhibitory control, and working memory, EF constructs that have been directly linked to academic achievement (McClelland and Cameron, 2019), and (2) the circumstances surrounding the COVID-19 pandemic made it impossible to work in close concert with schools and teachers to ascertain all of the learning materials being used by the individual participants, based on school year and age. Adolescents aged 11–18 were asked to take part with the plan of splitting them into younger and older age groups. An advantage of having adolescent participants is that it allowed for the study to be run independently online, without any researcher supervision. As Connolly et al. (2013) concluded that adolescents can both reliably and accurately report the noise acoustic levels within their environment and how it disrupts their learning, the current study had participants fill out a home environment and noise questionnaire. They were asked for specific details about their home and their subjective perceptions of the noise, which included measures of noise-induced annoyance. Based on their responses to the frequency of specific sound occurrences, they were then given an overall home noise score.

Participants then completed three different EF tasks: The Flanker, the Wisconsin Card Sorting Test (WCST), and the Backward Digit Span (BDS). The concurrent environmental noise being played through their headphones depicted a noisy and vibrant home, similar to what Connolly et al. (2019) had done with school noise, and following the previously mentioned review by Parmentier (2014), would represent the unpredictable and ever changing noise that is often present in a home and is most likely to cause distraction. The white noise was used to both serve as a constant background noise that was completely predictable and thus not distracting, and to block out actual environmental noise in the testing environment. The main experimental hypotheses were that overall, adolescents listening to the environmental noise would perform worse on all three EF tasks compared to their peers listening to the white noise. In terms of the effect of in-home noise on EF task performance, no specific hypotheses were predicted though these results were planned to be explored. It was, however, predicted that there would be an effect of experience with noise, whereby adolescents from quieter homes would have more difficulty on the tasks if listening to the environmental noise compared to their peers from noisier homes, who would have more practice in cognitively dealing with such distracting noise. Effects of age will be looked at, though exact predictions of the interactions with background noise and in-home noise were not made due to the lack of previous studies directly measuring this.

## Materials and Methods

### Participants

In total, 149 adolescents aged 11-to-18-years-old fully completed the online study from the comfort of their homes. As pre-registered, only the 129 who passed the headphone screening task (described below) who could ensure good audio quality were included. The mean age for these adolescents was 14.46 years (*range* = 11.08–18.92 years, *SD* = 2.11 years) with 74 females, 53 males, one gender fluid, and one not specified. To further ensure that the participants could appropriately see the visual stimuli presented on the screen and hear the auditory stimuli played through the headphones, they were asked about any visual or auditory impairments. Of these 129 participants, 101 reported no visual correction needed, 28 reported needing and wearing their corrective lenses, and none reported needing but not wearing their corrective lenses. Furthermore, 127 participants reported not needing a corrective hearing device, 2 reported needing and wearing their corrective hearing device, and none reported needing but not wearing their corrective hearing device. Thus, no participants were further excluded based on these criteria.

Data on participant ethnicity was collected and then grouped together based on the UK Office for National Statistics’ ethnic groupings^3^. For detailed demographic information, see Table 1. Participants were recruited using flyers seeking neurotypical adolescents between the ages of 11 and 18-years-old in the United Kingdom (*N* = 119) and United States (N = 10) via word of mouth, social media, online parenting groups, and a database of participants. Data collection occurred between 16 June 2020 and 11 April 2021. For a very detailed accounting of the COVID-19 responses and regulations within both the United Kingdom and United States, please see the following website^4^ describing the Oxford COVID-19 Government Response Tracker and its findings or see their published papers: United Kingdom- Cameron-Blake et al. (2020) and United States- Hale et al. (2020). Of note, all schools in both countries were closed for extended periods of time due to the pandemic, meaning that all participants in the current study experienced home-learning. Online written consent was obtained from each participant as well as from a caregiver, for those younger than 16 years of age. A £/$5 gift voucher was given to each participant to thank them for their time. This study was designed in accordance with the Declaration of Helsinki and reviewed and approved by the School of Sciences Ethics Committee at Birkbeck, University of London, reference number: 192071. The analysis plan for this project was preregistered on asprecited.org on the 5th of August 2020, reference number: 45752. Prior to this date, no data from this project was accessed or analyzed.

**TABLE 1:** Subject ethnicity by income-to-needs ratio.

		**INR Quartile Groups**			
**Ethnicity**	***n* (%)**	**1**	**2**	**3**	**4**
Arab	0	0	0	0	0
Asian or Asian British	6 (5.77)	3	0	2	1
Black or Black British	3 (2.88)	2	1	0	0
Mixed	10 (9.62)	5	1	2	2
White	85 (81.73)	16	35	12	22

*INR stands for income-to-needs ratio. INR quartile groups were created using the quartile cut offs of 16,875, 21,875, and 25,000. Frequencies of INR quartiles per ethnicity are reported above. Participant ethnicity was grouped. Arab was its own group. Asian or Asian British = Indian, Pakistani, Bangladeshi, Chinese, and any other Asian background. Black or Black British = Caribbean, African, and any other Black background. Mixed = White and Black Caribbean, White and Black African, White and Asian, and White and any other Mixed background. White = British, Irish, and any other White background.*

### Materials and Stimuli

The study was built and hosted on Gorilla Experiment Builder^5^. The executive function tasks were previously created on Gorilla to be used by experimenters. Participants completed the study via a link sent to them by the experimenter on a desktop computer or laptop device that was available to them. They were also asked to use any set of headphones that they had access to. Participants filled out two questionnaires and completed three executive function tasks.

### Sociodemographic-Short Questionnaire

The MacArthur Research Network on SES and Health (2008) Sociodemographic-short questionnaire was used to measure several facets of socioeconomic status. Slight changes were made to reflect both American and British culture. The questionnaire included two visual ladders of sliding scales, measuring subjective perspectives of one’s place within both the local community and the country. They were further asked about their highest level of education and their current job. In order to get an understanding of their income, they reported how much they earned in the past 12 months before deductions, how many adults bring income into the household, and how much total income they earned from all possible sources.

### Home Environment and Noise Questionnaire

This three-part questionnaire, using a 4-point Likert response scale, was created for the purpose of this study (see Supplementary Appendix A for the full questionnaire). Part one asked the participant about the make-up of their household, including dwelling type and number of inhabitants before the presence of COVID-19 (before March 1st, 2020) and during COVID-19 (after March 1st, 2020). The second part asked questions regarding subjective noise measures, including their annoyance to the noise in their work rooms as well as desired levels of noise for studying. The third section consisted of 25 questions asking about the frequency of specific noise sources in their homes. The questions themselves were designed so that half were positively stated, and the other half were negatively stated.

### Noise Recordings

Two different audio recordings were played through the headphones during the completion of the EF tasks. Audacity 2.4.1^6^ was used to put together the two audio recordings that made up the Environmental Noise and White Noise conditions. Individual sounds within the environmental noise recording were obtained from Freesound^7^ and included the following: airplane, vacuum, toilet flush, footsteps, washing machine, muffled T.V. (words not interpretable), gaming laser sound, dog barking, door opening and closing, doorbell ringing, traffic, birds, various toys, and children laughing. The white noise (pure noise 3) was downloaded from The MC^2^ Method online^8^. Both the White Noise and Environmental Noise recordings lasted for 15 minutes and were matched for frequency. A White Noise condition was used as a control to the Environmental Noise condition over silence as a means of blocking out the noises that would naturally be occurring in the participant’s homes during the completion of the task and would thus bias results.

### Headphone Screening

A headphone screening was used to (1) set the volume of the noise conditions, as we did not have direct control of the volume, and (2) to ensure the quality of the participant’s headphones. The screening task was developed in Gorilla Experiment Builder by Brown et al. (2018). Participants pressed a ‘play’ button on the screen that played a white noise track. They were instructed to set the volume to the “loudest level that you can tolerate the sound without feeling like it’s hurting your ears.” After this, participants were played three sounds which were specifically developed to only be distinguishable through headphones (i.e., they could not be appropriately distinguished through the computer’s speakers) and the participants were asked to determine if the first, second, or third sound was the quietest, as prompted on the screen. The correct answer was counterbalanced between being the first, second, and third tone played, with each repeated twice, giving a total of six trials. To pass the headphone screening, participants had to get five of the six trials correct. They moved onto the main tasks of the experiment once they passed and/or completed the three possible attempts. This allowed for all participants to have the chance to replace their headphones or to sort out any other issues before moving to the main tasks. The 19 participants that did not pass the headphone screening by the third attempt were not included in any analyses.

### Flanker Task

This task was developed in the Gorilla Experiment Builder by Anwyl-Irvine et al. (2020) based on the original task by Rueda et al. (2004). The Flanker task is an attention network test designed to measure inhibitory control. In the current experiment, participants were shown 5 arrows centrally on the screen. The middle arrow is referred to as the target arrow, with the surrounding arrows either appearing congruently and matching the direction of the central arrow or appearing incongruently and facing the opposite direction of the target arrow. The participants had to press the letter “z” on the keyboard if the target arrow was pointing to the left, or “m” if it was pointing to the right. They were asked to respond as quickly and as accurately as possible. The task began with 12 practice trials and feedback was provided for each trial informing them if they were correct or incorrect. The main task consisted of a total of 96 trials, which were separated into four blocks with a break in between. The participant had to press the spacebar to indicate that they were ready to begin the next block. For each trial, the arrows remained on the screen until the participant made a response. A central fixation cross appeared in between each trial with varying lengths of time (400, 600, 800, or 1000 ms). The task was counterbalanced in terms of the appearance of the central arrow (left or right) and the congruence of the surrounding arrows (congruent or incongruent). The trials and timings of the fixation cross were then randomized across participants. Dependent measures were based on reaction time (RT) and accuracy and are detailed in the results section.

### Wisconsin Card Sorting Test Task

This version of the WCST was attained and further developed from Gorilla’s task Samples^9^. This task was designed to measure set-switching and set-maintenance, utilizing abilities such as shifting, working memory, and inhibition (Huizinga and van der Molen, 2007). Each trial consisted of participants being given a target card to match with one of four other cards based on one of three rules: number (1, 2, 3, or 4), color (red, blue, green, or beige), or shape (circle, diamond, star, or triangle). This meant that three of the four cards to select from would each pair with the target card based on one rule alone, with the fourth card being a random card that did not match the target card. The task was designed to have 10 trials per rule block, with each rule repeating twice, giving a total of 60 trials. While the participants were aware of the three different rules, they were not told which rule to use nor when it would change. Therefore, they were only able to determine rule switches based on the 700 ms feedback they received after each trial. The rule block order for each participant was number, shape, color, shape, number, and color. The cards remained on the screen until the participant gave a response. There were a total of 64 unique cards which were pseudo-randomly displayed to ensure that there was no repetition of the target card, and that the different cards were spread out as non-target cards throughout and between the blocks. Dependent measures were based on errors made both within and between sets.

### Backward Digit Span Task

This task was created using the Gorilla Experiment Builder^10^ by Massonnié (2020), though minor adjustments were made to add our two auditory conditions. The digit span task is commonly used to study memory, with arguments made that the forward digit span task more specifically measures short-term memory while the BDS task measures working memory (Wells et al., 2018). Participants were shown a series of numbers and were asked to respond by inputting the same numbers in reverse order. The first level began with two numbers, with each new level increasing by one additional number. Each level contained five trials whereby the participant needed to get three of the five trials correct in order to advance to the next level. This meant that three mistakes within a level led to the termination of the task. Each trial began with a 450 ms fixation cross followed by each number presented one at a time on the screen for 1500 ms, with 500 ms intervals. The numbers were displayed in pseudo random order whereby each number was random other than that the same number could not directly follow the previously displayed number. Participants were first given two practice trials with feedback on their performance to help ensure that they understood the task. Dependent measures were the total number of correct trials (final score) and proportion of correct trials throughout the task.

### Procedure

Participants were first given information about the online study and, upon giving consent to participate, were then directed to begin. The study began by asking for the participant’s age and gender. To monitor the study and any potential issues, participants were also asked if any of their siblings had taken part and their age, and if they themselves had previously attempted to participate in the study but did not complete it. They were then asked to specify if their previous lack of completion was due to loading delays/poor connection, needing to stop for time reasons, or to state some other reason.

The parent was then instructed to complete the Sociodemographic questionnaire, followed by both the parent and the adolescent completing the Home Environment and Noise questionnaire. The adolescent was then asked to put their headphones on and to complete the headphone screening task, whereby they had three chances to pass, although all participants continued to the experimental portion of the study regardless of passing or failing. Upon completing the headphone screening, participants went on to complete three tasks: (1) the Flanker task, (2) the WCST task, and (3) the BDS task. Participants were randomly assigned to either the White Noise or the Environmental Noise condition. If in the Environmental Noise condition, they completed all three tasks while listening to an audio recording simulating a ‘noisy home environment,’ while if in the White Noise condition, they simply listened to an audio recording of white noise. The order of the tasks was randomized for each participant and the exact same audio recording, depending on noise condition, began playing at the beginning of each task and stopped once the task was completed, with the audio recording restarting each time. Therefore, they heard the same audio recording three times but for different lengths of time depending on the timing to complete each task.

Once the three tasks were completed, participants were then asked to state if the audio recording consistently played for the duration of each task, or if for any of the tasks the audio recording ended before the task was finished. They were then presented with a debrief of the study and were told that they had finished and could exit the browser window. The study took no more than 30 min to complete.

## Results

### Scoring and Preprocessing

#### Sociodemographic Questionnaire

Due to issues with collecting SES data through the study’s online format, which is explained in detail in the discussion, only total family income was looked at. As overall total income is not very informative when considering the complexity of socioeconomic status, it was therefore decided to report families’ income-to-needs ratio (INR). Total income was collected in bins, and INR was calculated by using the median of each income bin, similarly to King et al. (2020), and then dividing this number by the reported total number of inhabitants in the home before the pandemic. Calculated INRs were then grouped into quartile bins, with the break-down of ethnicity by INR quartiles seen in Table 1. While the current sample is perhaps slightly more heterogeneous than that often found within in-lab testing, it is still very much within the W.E.I.R.D. population. In terms of looking at income within later analyses, actual total income was used as income and number of inhabitants were individually investigated, and thus the combined INR measure was not used.

#### Home Environment and Noise Questionnaire

Responses to the 4-point Likert scale were added up to create an overall home noise score, where negatively phrased questions were reverse scored. The higher the overall score, the noisier the home was determined to be (lowest possible score = 25, highest possible score = 100). For part of the analyses, participants were grouped into noisier and quieter homes based on a median split (*Mdn* = 64.5).

#### Flanker

Two scores were pre-registered for this task. The Inverse Efficiency Score (IES) was developed to measure the participant’s ability to efficiently complete the task in terms of both timing and correct responses (IES = mean reaction time/proportion of correct trials), with higher scores meaning less efficiency. Following Imburgio et al. (2020), the mean reaction time to the incorrect trials was subtracted from the mean RT from the correct trials to get a ΔRT Accuracy score. A higher positive score indicates a bigger difference between the two trial types, with an average longer RT on correct trials and an average shorter RT on incorrect trials. The opposite direction for the correct and incorrect trials led to higher negative scores. Lower scores closer to zero infer that the reaction times to correct and incorrect trials are closer together and accuracy did not affect reaction time behavior. The congruency effect, here termed ΔRT Congruence, was further looked at as a measure of selective attention, whereby the mean reaction time on the incongruent trials was subtracted from the mean RT on congruent trials (van Leeuwen et al., 2007). Importantly, using these two difference scores allows for a better understanding of the effect of both accuracy and incongruency on performance, and removes the potential of simply looking at the effect of slow responders (Mullane et al., 2009).

As planned, four participants who reported audio issues during the task had their data removed. Furthermore, nine participants were excluded who did not pass the training (passing set at 8 out of 12 trials correct) and two whose performance was at chance level. All trials that were either less than 300 ms or greater than 1500 ms were removed (6.64% of total trials), and four participants with more than 25% of their data missing due to this criteria were excluded (van Leeuwen et al., 2007). After removing another two due to a combination of these issues, a total of 16.15% of the participants were removed from data analyses.

#### Wisconsin Card Sorting Test

As pre-registered, the WCST was scored based on errors made by the participant. Perseverative errors are those made based on following the rule from the previous set (does not apply to errors made in the first block), while non-perseverative errors are all other errors made. Of note, the first error made after a rule change is not counted as a perseverative error but as a non-perseverative error, as this is the first instance that the participant learns that the rule has changed. Any error after this that is made based on the previous rule set would then count as a perseverative error. The last score was failure to maintain set, established as the participant making an error after having gotten at least five correct in a row, all within the same rule block. The last score included was total errors made. Importantly, a single error made could be allocated toward one or more of the scores. For an overview of WCST scoring, see Cianchetti et al. (2007).

As planned, trials with a response time that exceeded 10s were removed (0.016% of all trials) (Piper et al., 2012). Although not planned, the nature of the task meant that those with worse internet connections experienced severe loadings delays. Additionally, several participants had long gaps in between trials. As both issues would strongly interfere with the participant being able to follow the rule sets, it was objectively determined to remove the data from four participants who took longer than two standard deviations above the mean to complete the task (*M* = 3.27 min, *2 SD* = 6.65 min), of which three of these participants experienced loading delays. No participants reported any audio issues, and only a total of 3.08% of participants were excluded.

#### Backward Digit Span

Both scores used were pre-registered. Final score is a commonly used measure (e.g., Lipsey et al., 2017) and represents the total number of correct trials. As participants could have achieved the same final level with either two errors per level or with none until the final level, we further looked at the proportion of correct trials to account for this difference.

Data from three participants were excluded as they did not follow the rules of the task (reported the numbers in forward order). As planned, data was excluded for those with audio issues, with 13 participants who had audio issues during the task and seven who self-reported having audio issues. A further three participants were excluded due to a combination of these issues. In total, 20.00% of participants were excluded.

### Analyses

#### Home Environment, Subjective Noise Measures, and Executive Function

As pre-registered, analyses were performed to capture an understanding of the home environment, and how it may be affecting adolescents. As can be seen in Table 2, the number of inhabitants in the home during the pandemic both increased and decreased compared to the number of inhabitants before the pandemic hit. A paired-samples *t*-test revealed a small, though significant overall increase from the number of inhabitants occupying the home before the pandemic (*M* = 3.97, *SD* = 1.11) to during the pandemic (*M* = 4.10, *SD* = 1.22), *t*(127) = –2.79, *p* = 0.006. Although there was a significant difference in the number of inhabitants before and during the pandemic, only the number of inhabitants during the pandemic was used in the following correlations as this number would be more representative of the adolescents’ home environment when answering the questionnaires. Furthermore, though our sample was skewed toward participants from the United Kingdom, country of residence did not significantly correlate with any of the home or subjective noise measures, meaning that our sample did not significantly differ in the recorded home measures nor the subject noise measures across country of residence. Spearman bi-variate two-tailed correlations were run looking at the home environment and subjective noise measures (see Table 3). Correlations were Bonferroni corrected and significance was established at 0.00625 (0.05/8).

**TABLE 2:** Frequency of the type of home and the number of inhabitants in the home before and during the pandemic.

	***n***	**%**	**% Change**	**Mean**
**Type of home**	128			
Detached	35	27.34		
Collective dwellings	93	72.66		
Semi-detached	24	18.75		
Terraced	33	25.78		
Flat	36	28.13		
**Number of inhabitants before**	127			3.99
2	3	2.36		
3	38	29.92		
4	57	44.88		
5	21	16.54		
6	6	4.72		
7	0	0.00		
8	1	0.79		
9	0	0.00		
10	1	0.79		
**Number of inhabitants during**	127			4.13
2	3	2.36	0.00	
3	35	27.56	−2.36	
4	54	42.52	−2.36	
5	21	16.54	0.00	
6	11	8.66	3.94	
7	0	0.00	0.00	
8	2	1.57	0.79	
9	0	0.00	0.00	
10	1	0.79	0.00	

**TABLE 3:** Correlations between participant age, home measures, and subjective noise measures.

	**1**	**2**	**3**	**4**	**5**	**6**	**7**	**8**	**9**
(1) Age	1	–0.223	0.207	–0.135	0.026	0.07	0.023	0.013	0.167
(2) Total income		1	–0.2	0.058	–0.164	0.057	0.042	–0.218	–0.072
(3) Home noise score			1	0.051	0.243*	0.123	0.261*	0.348*	0.240*
(4) Number of inhabitants				1	−−0.291*	0.174	–0.142	–0.11	0.143
(5) Dwelling type					1	–0.029	–0.043	0.18	0.019
(6) Comparative noise annoyance						1	–0.106	0.05	0.309*
(7) Studying noise preference							1	0.095	0.094
(8) Room noise level								1	0.351*
(9) Room noise annoyance									1

*Age and home noise score were entered as continuous variables, all other variables are ordinal. N = 106–128*

*p < 0.00625.

Of note, age did not significantly correlate with any of the home measures nor the subjective noise measures, meaning that younger adolescents were not more sensitive to noise, did not perceive more noise, nor were they more annoyed by noise than their older peers. Those who reported being more annoyed by the noise in the room they study in were significantly more likely to report higher annoyance to noise compared to their peers, and to be studying in a noisier room. Furthermore, number of inhabitants was also found to significantly correlate with dwelling type, with more inhabitants in the home being more likely to live in less collective dwellings. Higher home noise scores significantly correlated with more collective dwelling types and correlated with adolescents reporting more noise in the room they study in.

Interestingly, the correlations further revealed that those from noisier homes were more likely to report a preference for more background noise when studying while also being more annoyed by the noise in the room they study in; however, noise preference and noise annoyance while studying did not correlate. To further understand this seemingly contradictory finding, further analyses were done to determine if perhaps those from noisier homes are either likely to develop a preference to noise based on their exposure to it, *or* to become more annoyed by it. After grouping participants into noisier and quieter homes, however, those from noisier homes did not show the expected negative correlation, meaning that those who reported a preference for noise did not also report less noise annoyance, and vice versa.

Further spearman correlations were run to determine the relationship of task performance with both home measures and subjective noise measures (see Table 4). Correlations were Bonferroni corrected for multiple comparisons, with home measures being significant at 0.0166 (0.05/3) and subjective noise measures being significant at 0.0125 (0.05/4). Again, country of residence did not correlate with EF task performance. Results revealed that measures of the home did significantly relate to task performance. More inhabitants in the home during the pandemic significantly related to more perseverative errors and total errors on the WCST, and nearly significantly related to a higher Flanker ΔRT Accuracy score. Interestingly, being in more of a collective dwelling significantly correlated with a lower BDS final score but near significantly correlated with less total errors on the WCST. A more collective dwelling also nearly correlated with a better Flanker IES and significantly correlated with a lower Flanker ΔRT Accuracy score. As for the relationship between subjective noise characteristics and task performance, only a higher reported annoyance to noise correlated with less WCST failures to maintain set.

**TABLE 4:** Correlations of task scores with home measures and subjective measures of noise.

	**Home measures**			**Subjective noise measures**			
	**Total income**	**Number of inhabitants**	**Dwelling type**	**Comparative noise annoyance**	**Studying noise preference**	**Room noise level**	**Room noise annoyance**
(1) Flanker IES	0.081	–0.011	**−−0.215^†^**	0.047	–0.032	0.070	–0.035
(2) Flanker ΔRT accuracy	0.065	**0.223^†^**	−−0.264*	0.008	–0.178	–0.149	–0.121
(3) Flanker ΔRT congruency	0.033	–0.025	0.062	–0.081	0.050	–0.045	–0.118
(4) WCST perseverative errors	–0.137	0.164	–0.138	0.036	–0.050	0.037	0.157
(5) WCST non-perseverative errors	0.030	0.349*	–0.177	0.090	–0.065	–0.181	0.139
(6) WCST set failure	0.089	–0.075	–0.041	–0.040	0.073	–0.042	−−0.264*
(7) WCST total errors	–0.060	0.296*	**−−0.194^†^**	0.069	–0.098	–0.104	0.170
(8) BDS final score	0.130	0.141	−−0.248*	–0.006	–0.058	–0.168	–0.109
(9) BDS proportion correct	0.021	0.147	–0.125	–0.118	0.005	–0.118	–0.047

*All home measures and subjective noise measures are ordinal, other than the number of inhabitants. Total income has a scale of 1 to 9, while all other ordinal measures have a scale of 1 to 4. *N* = 82–125.*

*Significant.

*^†^Near significant.*

#### Experimental Noise and Executive Function Task Performance

According to plan, participant task scores and the noise questionnaire scores that were above or below three standard deviations from the mean were removed before analysis (fewer than four data points were removed for each variable). See Table 5 for the included ages and genders across experimental conditions and home noise groupings. Following the same plan, order effects from background noise habituation were also checked, since participants heard the same noise soundtrack during each task. Task order and noise condition were run through a MANOVA which included all task scores. There were no main effects of noise condition nor any significant interactions between task order and noise condition. The only main effect of task order was for the Flanker IES, *F*(2,75) = 5.45, *p* = 0.006, η^2^_p_ = 0.125. A Tukey *post hoc* test revealed that those who completed the Flanker task as their second task had a significantly higher IES score (*M* = 629.36, *SD* = 110.83) than those who completed it as their first (*M* = 554.54, *SD* = 100.89, *p* = 0.012) or third (*M* = 551.01, *SD* = 79.56, *p* = 0.018) task. There was no significant difference in IES between those who completed the Flanker task first or last. Thus, regardless of being in the White Noise or the Environmental Noise condition, those who completed the Flanker task second seemed to be less efficient at completing the task than those who completed the Flanker task first or last.

**TABLE 5:** Breakdown of subject characteristics within both experimental Noise Condition and Home Noise grouping based on gender and age.

	**Noise Condition**				**Home Noise**				**Total Sample**	
	**White**		**Environmental**		**Quieter**		**Noisier**			
	***N***	***M***	***N***	***M***	***N***	***M***	***N***	***M***	***N***	***M***
**Gender**	38		42		38		42		80	
Female	18		26		18		26		44	
Male	20		16		20		16		36	
**Age (years)**	39	14.64	43	14.76	39	14.34	43	15.03	82	14.70
11	7	11.55	4	11.48	9	11.50	1	11.62	11	11.52
12	3	12.33	6	12.46	5	12.25	4	12.62	9	12.42
13	6	13.42	4	13.58	4	13.33	6	13.58	10	13.48
14	3	14.17	10	14.35	5	14.42	8	14.24	13	14.31
15	7	15.27	4	15.42	4	15.54	7	15.20	11	15.33
16	7	16.22	6	16.18	4	16.27	9	16.17	13	16.20
17	1	17.67	8	17.43	5	17.47	4	17.44	9	17.46
18	5	18.38	1	18.08	3	18.19	3	18.47	6	18.33
**Age groups**										
Younger (11–14)	19	12.68	24	13.27	23	12.62	20	13.46	43	13.01
Older (15–18)	20	16.50	19	16.65	16	16.82	23	16.40	39	16.57

With no significant habituation to the noise found, as pre-registered, the effects of noise condition, home noise scores and age on task performance were looked at. A 2 (noise condition: white, environmental) × 2 (home noise: quieter, noisier) × 2 (age: 11–14, 15–18) MANCOVA was run looking at all task scores, with all predictors being between-subject variables. A multivariate analysis was run instead of the planned separate univariate tests for each task score to enable equivalent sample sizes and participants be included in each analysis, ensuring comparability between the findings. Although country of residence (United Kingdom or United States) did not significantly correlate with the home measures, subjective noise measures, and task performance, many cultural differences could still be present that were not accounted for that could interact with the effect of noise on EF task performance. Thus, country of residence was added into the analysis as a covariate. Changes to the SPSS syntax were made (see Supplementary Appendix B) in order to use the covariate influenced adjusted means in the *post hoc* analyses, and all pairwise comparisons were Bonferroni corrected. No significant effect of country was found.

##### Flanker

**FIGURE 1:**
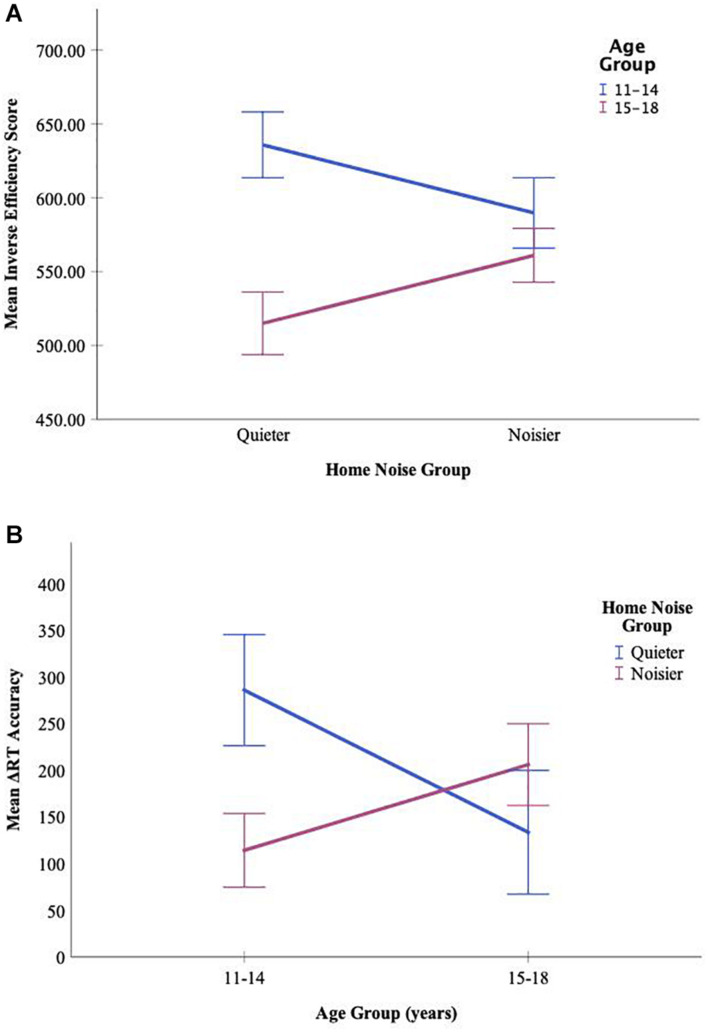
Depiction of the interaction between the age groups and the home noise groups on performance in the Flanker task. Age groups were split into a younger group (ages 11–14) and an older group (ages 15–18), and home noise groups were based on the median split of the home noise scores derived from the Home Environment and Noise Questionnaire (*Mdn* = 64.5). The bars in the graph represent standard error. **(A)** When looking at those participants from quieter homes, older adolescents were significantly more efficient compared to their younger peers, though when looking at those from noisier homes, older participants were no longer performing better than their younger peers. **(B)** For younger adolescents, those from quieter homes had significantly higher ΔRT Accuracies compared to their peers from noisier homes. There was no difference in performance between the older peers in terms of home noise levels.

The Flanker task consisted of IES, ΔRT Accuracy, and ΔRT Congruency scores. Results revealed no main effects of noise condition or home noise, but there was a significant main effect of age for the IES score, with older participants (*M* = 533.20, *SD* = 88.21) having a more efficient score than the younger participants (*M* = 612.52, *SD* = 107.91), *F*(1,73) = 12.02, *p* = 0.001, η^2^_p_ = 0.141. There were no significant interactions between noise condition and home noise or age; however, the IES score had a significant interaction between home noise and age, *F*(1,73) = 5.50, *p* = 0.022, η^2^_p_ = 0.070. Further analyses revealed that if from a quieter home, the older adolescents performed more efficiently (*M* = 509.71, *SD* = 84.76) than the younger adolescents (*M* = 641.08, *SD* = 106.70), *p* < 0.001, but this advantage was no longer present if they were from a noisier home (*M_15–18_* = 556.69, *SD*_15–18_ = 87.36 and *M*_11–14_ = 583.96, *SD*_11–14_ = 106.61), *t*(41) = 0.97, *p* = 0.34 (see Figure 1A).

Furthermore, the ΔRT Accuracy score had the same significant interaction between home noise and age, *F*(1,73) = 3.97, *p* = 0.050, η^2^_p_ = 0.052. Further analyses revealed a different direction, however, whereby in the younger age group, adolescents from quieter homes had a higher ΔRT Accuracy (*M* = 285.72, *SD* = 285.42) compared to their peers from noisier homes (*M* = 132.85, *SD* = 176.67), *p* = 0.045. There was no difference though in ΔRT Accuracy depending on home noise in the older adolescents (*M*_*quieter*_ = 146.33, *SD*_*quieter*_ = 265.16 and *M*_*noisier*_ = 209.79, *SD*_*noisier*_ = 210.28), *p* = 0.42 (see Figure 1B). Lastly, there were no significant three-way interactions between noise condition, home noise, and age for the Flanker task.

##### Wisconsin Card Sorting Test

The WCST was scored based on the number of perseverative errors, non-perseverative errors, failure to maintain set, and total errors. The analyses revealed no main effects of noise condition, home noise, or age. While there were no significant interactions between home noise and noise condition or age, there were two near significant interactions between noise condition and age: Perseverative errors [*F*(1,73) = 3.23, *p* = 0.076, η^2^_p_ = 0.042] and total errors [*F*(1,73) = 3.15, *p* = 0.080, η^2^_p_ = 0.041]. The younger adolescent group showed a trend toward more perseverative errors in the Environmental Noise condition (*M* = 5.52, *SD* = 4.72) compared to those in the White Noise condition (*M* = 3.08, *SD* = 2.25), *p* = 0.064; however, there was no difference between the noise conditions for the older adolescents (*M*_*enviro*_ = 4.26, *SD*_*enviro*_ = 3.99 and *M*_*white*_ = 5.19, *SD*_*white*_ = 4.73), *p* = 0.49. Similarly, the younger adolescents also trended toward making more total errors in the Environmental Noise condition (*M* = 20.12, *SD* = 11.55) than in the White Noise condition (*M* = 15.09, *SD* = 5.32), *p* = 0.088. Again, there was no difference in total errors made between the two noise conditions for the older adolescents (*M*_*enviro*_ = 15.97, *SD*_*enviro*_ = 6.68 and *M*_*white*_ = 18.38, *SD*_*white*_ = 10.38), *p* = 0.43. Finally, no three-way interactions were found.

##### Backward Digit Span

The BDS task was evaluated based on final score and proportion correct. Analyses revealed no significant main effects or interactions.

## Discussion

The main purpose of the current study was to run an independent online experiment looking at the effect of the home, and in particular in-home noise, on adolescent EF. The EF tasks used reflect the skills that are frequently needed within academic learning; therefore, any effects on their EF task performance could indicate the potential impact that noise may be having on their in-home learning during the pandemic. Another more exploratory avenue of the current study was to understand how factors determining the home environment relate to subjective perceptions of noise, and how these both might relate to adolescent EF.

### Correlations Between the Home Environment, Perception of Noise, and Executive Function

In terms of the home environment, it is clear that the pandemic led to population shifts. The recorded decreases and increases of inhabitants in the home could represent both the more vulnerable inhabitants moving out of the home to be more protected on their own, as well as separate households grouping together to support each other throughout the pandemic and ongoing lockdowns. While the specific reasons for shifting homes were not directly recorded, overall, there was a small but significant increase in the number of inhabitants living in the home during the pandemic compared to before, indicating that the core make-up of a home was affected by the pandemic.

To get a better understanding of the adolescents’ home environments, the current study further measured total family income, in-home noise levels, number of inhabitants, and dwelling type. The home noise score that was derived from the questionnaire positively correlated with dwelling type, indicating that the more collective the dwelling, the higher their in-home noise scores. While we did not directly measure noise levels in the participants’ homes, this finding does follow the same conclusion as Pujol et al. (2012) who directly measured the in-home noise levels of a similar demographic (20% detached dwellings and 80% collective dwellings) over 8 days. It was further found that more inhabitants in the home also coincided with living in less collective dwellings, consistent with larger families needing a bigger home. What is interesting, though, is that unlike Pujol et al. (2012), a correlation between in-home noise levels and number of inhabitants was not found. While we cannot conclude from the current results that more people dwelling together during the pandemic increased the noise levels, having a more direct measure of home noise and any changes in the noise levels from before the pandemic to during the pandemic, along with the shifts in household numbers, might better capture this relationship. Another divergence from previous literature (Dale et al., 2015; Casey et al., 2017) was found, where a lower income did not correlate with higher in-home noise levels. Perhaps a more concise depiction of SES, as was originally planned in the study, would have been better able to measure this.

Subjective perceptions of noise were also recorded, including adolescents’ general annoyance to noise compared to their peers, their preference for background noise when studying, their perception of the noise level in the room they study in, and their annoyance with the in-room noise. The two significant correlations between these were that the higher they reported their annoyance with in-room noise, the noisier they reported their room to be and the more annoyed to noise in general they reported being compared to their peers. This coincides with the literature, where higher noise levels correlated with higher rates of annoyance (Lundquist et al., 2000; Ali, 2013; Minichilli et al., 2018). In terms of how the home measures correlated with subjective noise measures, unsurprisingly, higher home noise scores correlated with a perception of higher in-room noise levels. Furthermore, those with higher in-home noise scores were more likely to report a preference for a noisier background environment when studying, and also more annoyance with in-room noise. Evidence for the possible explanation that those from noisier homes either develop a preference for noise *or* become more annoyed by noise, was not found. Thus, the findings suggest that those from noisier homes both prefer to have more noise in the background when studying yet are also more annoyed by in-room noise. Perhaps, those from noisier homes find it more difficult to work in silence and require some noise in the background to match the environment that they are most used to, but that these same noisier homes are more likely to have particular sound sources that are more annoying than would be found in a quieter home. This would align with Connolly et al.’s (2013) finding where adolescents reported different levels of annoyance depending on the type of sound present, meaning that further research into the varying effects of specific noise sources within the home is needed. Of note, as the home noise score was based on the reporting of the frequency of specific in-home sounds, this score is therefore susceptible to subjective perceptions of noise and thus it is not surprising that the noise score correlated with subjective noise measures.

Contrary to the literature showing an effect of annoyance by age within school environments (Ali, 2013; Connolly et al., 2013; Minichilli et al., 2018), the current study did not find a difference in home noise-induced annoyance in younger versus older adolescents. However, the cited studies took place within school settings, and not within a home-learning environment during a pandemic. There are many possible reasons for which annoyance levels might now differ, from familiarity with home noises to frustration with trying to learn in novel circumstances. Task type and cognitive demand at the time of reporting have also been suggested to mediate the effect of age on noise annoyance (Connolly et al., 2013). Lastly, as age did not correlate with the home measures, we can conclude that while our age range was large, participants within each age-point included came from diverse homes, strengthening the generalization of our findings. With the current evidence that reports of noise-induced annoyance relate to dwelling type and do not relate to age, it is clear that the findings on annoyance from the school literature cannot fully capture what is happening in the home and how adolescents are being impacted by in-home noise during the pandemic.

We further looked at how measures of the home environment and subjective measures of noise might relate to adolescent performance on the three EF tasks. While total family income did not correlate with task performance, number of inhabitants in the home during the pandemic related to both the Flanker and the WCST tasks. Adolescents in a home with more inhabitants trended toward having a higher Flanker ΔRT Accuracy score and had significantly more non-perseverative and total errors on the WCST. Furthermore, those adolescents who live in a more collective dwelling trended toward being more efficient on the Flanker task and having significantly lower ΔRT Accuracies. They also trended toward being more likely to have fewer overall errors on the WCST and were significantly more likely to have a worse BDS final score. While it appears that overall a higher number of inhabitants correlates with worse performance on the WCST- a task that involves shifting, working memory, and inhibition- and a potential difference in response time behavior on the Flanker inhibitory task, the relationship with dwelling type is not as clear.

It seems that while there is evidence that being from a more collective dwelling positively correlates with better task performance on the Flanker and WCST, this also negatively correlates with performance on the BDS task. However, as will be discussed later, data from the BDS task may not be reliable and thus might explain this conflicting finding. This might then infer that overall, coming from a home with closer and more neighbors may be linked to better adolescent EF. Lastly, in terms of subjective measures of noise and EF task performance, being more annoyed by in-room noise correlating with less set failures on the WCST was the only significant result. While the direct relationship between EF task performance and both measures of the home environment and subjective noise cannot be inferred, it is clear that factors strongly determining the home environment are linked to adolescent EF abilities; the link between individual differences in the subjective experience with noise and EF is less evident.

### Effect of Noise on Executive Function

It was hypothesized that there would be a direct effect of the audio recording condition on task performance. While we did not find this overarching effect, when splitting participants into younger and older adolescent age groups, results showed a clear *trend* on the WCST whereby the younger adolescents were making more perseverative and total errors in the presence of the environmental noise, compared to those simply in the white noise condition. Connolly et al. (2019) did find a significant interaction of age and school environmental background noise, though the study specifically looked at reading ability and found varying age effects at different noise levels. While the current results just missed statistical significance, the evident and identical direction of the trends mean that while a strong conclusion cannot currently be made, nor can these results be discounted. Future research should look at how changing and dynamic sounds often found in noisier homes directly impact on learning.

A main effect of age was found when looking at the Flanker task efficiency score, which takes into account speed of reaction time and accuracy, with older adolescents performing more efficiently than their younger peers. Furthermore, while there was not a clear prediction for the effect of the in-home noise on task performance, when splitting the participants into their separate age groups, we did find significant results for this same Flanker efficiency score. When looking at those from quieter homes, older adolescents still demonstrated more efficiency on the Flanker task than their younger peers, but this advantage disappeared when looking at those from noisier homes. The overall finding that older adolescents perform more efficiently on this EF task regardless of noise follows previous research (for a review, see Ridderinkhof et al., 2021). What is, however, unexpected and remarkable, is the finding that when taking into account individual differences, such as the noise levels that the adolescent experiences on a daily basis at home, the older adolescents no longer show this developmental advantage in their performance on the task.

Another interaction between the effects of in-home noise and age on the Flanker task was found for the ΔRT Accuracy score. When looking exclusively at the younger adolescents, those who came from noisier homes had higher ΔRT Accuracy scores compared to their peers from noisier homes. This implies that if they experience more in-home noise, they are more likely to have similar reaction times for both correct and incorrect trials, whereas those from quieter homes clearly have a behavioral difference in their response times depending on accuracy. With no differences found for the older adolescents, it is apparent that only the younger adolescents are impacted by the long exposure to noise in this instance.

Overall, we can infer from these findings that regardless of the noise recording being played during the experiment, the noise that adolescents are frequently surrounded by in their home is having long-term effects on their EF. This finding, therefore, extends past pandemic-specific circumstances as it implies that regardless of the environment that they are learning in, be it their home or their school, coming from a home with higher noise levels can have disadvantageous effects for both older and younger adolescents.

Of note, it was predicted that there would be an interaction between noise condition and home noise, where those who experience higher in-home noise on a daily basis would do better in the environmental noise condition than those from quieter homes; however, no evidence was found for this on any of the tasks. Therefore, it does not seem that experience with in-home noise translates to a novel learning situation with similar noise. One potential limitation of the study that could explain why there was not an effect of audio recording condition on task performance could be that since participants heard the same audio recording repeated for each task, over time, they could have habituated to the noise and thus their performance would no longer have been affected by it. However, no order effects based on noise condition were found, meaning that the participants did not become habituated to the noise. Another possible explanation is that while the environmental audio was created to depict a naturalistic noisy home, homes can vary on specific sounds sources and frequency of sounds; thus, perhaps these intricacies that make up their in-home noise experience need to be matched in the audio in order for them to perform better compared to their peers from quieter homes. For example, while some participants from a noisy home may frequently hear planes overhead, peers from equivalently noisy homes may never hear planes, and thus would get more distracted by this sound source while completing the tasks. Thus, as mentioned previously, further research into the varying impact of specific sound sources within the home is needed.

Going back to the results on order effects, an overall effect of task order regardless of background noise was uncovered, with a higher IES score when the Flanker task was completed second. This potentially could be explained by research showing “inhibitory fatigue,” whereby when completing two consecutive inhibition tasks, performance on the second is likely to be poorer than if a different task had preceded it (Diamond, 2013). However, because the WCST preceded the Flanker task both when the Flanker was completed second and third, the finding of decreased efficiency when completed second cannot be due to this. As the order effect found did not interact with background noise, age, and home noise when these were checked, while there is no clear explanation for the finding, it was concluded that it had no influence on the current findings.

### Limitations and Future Directions

As the current study was designed and completed during a pandemic, it is important to highlight the limitations that were present in the current design. Importantly, while the two noise conditions used offered the ability to understand the influence of environmental noise, a true control condition without any noise would have been preferable. For instance, Helps et al. (2014) covaried for performance in a no-noise condition to determine the true effect of different levels of white noise on performance when testing children in a school room setting. While this may be feasible for certain designs where the children are all tested in the same environment and are exposed to the same environmental noise in the room, this was not feasible to implement in the current study. It is important to note as well that white noise has been found to influence children’s EF task performance, with certain levels of white background noise aiding low-attentive children and hindering high attentive children (Söderlund et al., 2010; Helps et al., 2014). Future research looking at the differences between environmental noise, white noise, and no noise would help to better understand and interpret the current findings. Additionally, the noise questionnaire used here has not been validated against true measures of in-home noise levels, and as previously mentioned, it is susceptible to subjective perceptions of noise. Without a direct measure of noise, the current study was not able to disentangle objective and subjective effects of noise, though with learning being such a multifaceted construct, it is likely that both play an important role. In terms of the participants, while neurotypical adolescents were advertised for during recruitment, further checks should be implemented in future to ensure that other factors linked to EF ability and noise sensitivity, such as autism (Kouklari et al., 2018; Schwartz et al., 2020), are not influencing the results.

Of further note, as the EF tasks used were a proxy for the cognitive demands often found within academic learning, the current study is a first step toward understanding the direct effect of in-home noise on home-learning. Further research is very much needed to fully understand the extent to which the pandemic has affected students within secondary education. For instance, a recent study by Muzi et al. (2021) looking at adolescent wellbeing during the pandemic found an increase in problematic social media usage, which was then further linked to higher rates of attentional and other emotional-behavioral problems. Future work should therefore look at the interplay between noise and social media distractions and its effects on adolescent attention, EF, and learning, especially within the context of the pandemic. The authors further highlight how adolescents with insecure attachment may be more susceptible to the fear and isolation brought about by the pandemic (Muzi et al., 2021). With attachment being linked to both EF (Escobar et al., 2013) and the home environment (Klemfuss et al., 2018), it would be important to take into account how attachment may be moderating the relationship between in-home noise and EF task performance, particular when considering that certain social-induced noises (e.g., a parent scolding a sibling) may have a different effect and may be more linked to attachment than a non-social noise (e.g., the washing machine running).

### Advantages and Disadvantages of Independently Run Online Research

The potential advantages of independently run online research is vast, both for researchers and for the inclusion of heterogeneous participants. Importantly, for researchers, independent online research can increase productivity by reducing the many months, and sometimes years that are spent collecting data. In addition, it allows for research groups with less funding for bringing participants into the lab, or indeed smaller spaces, to conduct large-scale projects. Furthermore, projects and ideas are sometimes limited due the time imposed by data collection, and an increase in the online tools available to conduct high caliber research can significantly change this. Crucially though, there are certain tasks and forms of research that will not be able to be translated to an online and/or independent format. The BDS task used in the current study is a prime example. While it is a popular and well validated working memory task within the field due to its use in the standardized Wechsler Intelligence Scale for Children (Wechsler, 2014), it does not translate well to an online and independent format. Regardless of telling participants to not write down the numbers, it is likely that many participants still did this, potentially explaining the current lack of findings for this task. Thus, as the BDS does not seem to be adaptable, conclusions for BDS performance have not been made in the current study. With no easy way of controlling for this limitation, the future use of this task in independently run online environments is not advised.

In terms of online research helping with participant heterogeneity, as recruitment is not limited to a specific location, it has the potential to recruit a much more diverse participant pool. Location based research tends to only attract families of higher socioeconomic status that have the time and financial freedom to travel and spend a few hours at the lab, making it difficult to break the W.E.I.R.D cycle of data collection. Running independent online research can also enable more global research, as time zones are no longer a constraint. Of note though, simply translating research to an online format does not automatically lead to a more heterogeneous sample, as can be seen in the current sample, and careful steps still need to be taken to include a more diverse sample. Furthermore, issues with collecting sociodemographic information arose. In the current study, it was evident that these independent adolescents occasionally completed the sociodemographic questionnaire with their own information rather than their parents’, reducing the data that we could interpret. We did find that including the option to select “Do not know,” as we had for total family income, reduced the reporting of incorrect data. Thus, by making it abundantly clear who the question is referring to, as well as giving participants an option to opt out in case their parent is not accessible at the time of completing the questionnaire, will ensure accurate sociodemographic data collection.

Naturally, with an independently run online study, there is less researcher control over the testing environment. Steps, however, can be taken to ensure experimental rigor. For instance, as auditory stimuli were key for the current experiment, an objective headphone screening task worked well to guarantee good hearing ability, working headphones, and that the participants were wearing the headphones. This did, however, mean that before data processing, 19 participants were already excluded. Furthermore, additional pre-processing steps were included in the current study to help ensure high data quality. Participants or individual data points were excluded based on loading delays, time taken to complete the task, response time, not following the rules, audio consistently restarting, audio stopping before the end of the task, and self-reported audio issues. Unfortunately, this inevitably means greater data loss, with many of these exclusions not being necessary or as common during in-lab testing. Fortunately, with online data collection being faster, including more participants is easy enough to ensure high data quality. So while some control of the testing environment is lost in online and independently run studies, steps can be implemented to resolve these issues and allow researchers to reap the many benefits that this methodology enables.

## Conclusion

Overall, the current study clearly demonstrates that the home environment influences the subjective perception of noise. In particular, we found converging evidence with the school literature that higher levels of noise correlate with higher rates of annoyance in adolescents. Furthermore, while we did not find a significant direct effect of background noise on EF task performance, actual in-home noise levels significantly affected task performance. Regardless of the background audio presented while completing the task, both younger and older adolescents showed evidence that consistently being in a noisy home impacted their EF task performance. With in-home noise levels having long-term effects on EF, it is clear that more research needs to be done to better understand the influence that the home environment may be having on learning within the home, as well as within schools.

## Data Availability Statement

The raw data supporting the conclusions of this article will be made available by the authors, without undue reservation.

## Ethics Statement

The studies involving human participants were reviewed and approved by the School of Sciences Ethics Committee at Birkbeck, University of London (reference number: 192071). Written informed consent to participate in this study was provided by the participants’ legal guardian/next of kin, or by the participant themself if they were 16 years of age or older.

## Author Contributions

BC and NK contributed to the conception and design of the study. BC created the study, recruited participants, pre-processed the data, performed the statistical analysis, and wrote the first draft of the manuscript. NK wrote sections of the manuscript. Both authors contributed to manuscript revision, read, and approved the submitted version.

## Conflict of Interest

The authors declare that the research was conducted in the absence of any commercial or financial relationships that could be construed as a potential conflict of interest.

## Publisher’s Note

All claims expressed in this article are solely those of the authors and do not necessarily represent those of their affiliated organizations, or those of the publisher, the editors and the reviewers. Any product that may be evaluated in this article, or claim that may be made by its manufacturer, is not guaranteed or endorsed by the publisher.

## Footnotes

1
unicef.org

2
epa.gov

3
www.ons.gov.uk

4
https://www.bsg.ox.ac.uk/research/research-projects/covid-19-government-response-tracker

5
www.gorilla.sc

6
www.audacityteam.org

7
www.freesound.org

8
www.mc2method.org

9
https://gorilla.sc/support/samples

10
https://gorilla.sc/openmaterials/36699
