# Supplementary material for: The Negative Impact of Noise on Adolescents’ Executive Function: An Online Study in the Context of Home-Learning During a Pandemic
Source: Front Psychol. 2021 Sep 22;12:715301. doi: 10.3389/fpsyg.2021.715301 (PMC8492971; doi:10.3389/fpsyg.2021.715301)
Supplement: Supplementary file 1 [file Image_1.pdf]

Appendix A

The full Home Environment and Noise Questionnaire that all participants filled out.

Home Environment and Noise Questionnaire

The purpose of this questionnaire is to get an understanding of your home environment. Please answer the following questions to the best of your ability. You can skip over any questions that you do not feel comfortable answering.

PART 1:

1) How many people were consistently living in your home before the COVID-19 pandemic (before March 1st)?

    Number of adults in our home: _______

    Number of children in our home: _______

2) How many people have been consistently living in your home since the COVID-19 pandemic began (since March 1st)?

    Number of adults in our home: _______

    Number of children in our home: _______

3) What type of home do you have?
    a. Detached house: a single unit house that does not contain a directly connected house.
    b. Semi-detached house: a single unit house that is directly connected to another house on only one side.
    c. A terraced house: a single unit house that is directly connected to another house on both sides.
    d. A flat: a single residence in a building that contains either upstairs and/or downstairs neighbours.

PART 2:

4) For the following 3 questions, please think of the room that you have been studying the most in while completing your schoolwork from home during the pandemic:
    a. On average, how quiet or noisy is this room?

1	Very quiet	Somewhat quiet	Somewhat noisy	Very noisy	4

    b. On average, how annoyed do you get from the noises that you hear in this room?

1	Never Annoyed	Sometimes Annoyed	Frequently Annoyed	Always Annoyed	4

    c. What sounds do you notice in this room that cause you the most annoyance and distraction? Please list them below.

    ____
    ____
    ____
    ____

5) When completing your schoolwork, what level of noise do you prefer to have in the background? (Example: do you prefer complete silence, music in the background, or noises from the outdoors in the background?)

1	Very Quiet	Somewhat Quiet	Somewhat Noisy	Very Noisy	4

6) How would you rate how annoyed you get towards sounds/noises in general compared to other people?

1	Much less easily annoyed	Somewhat less easily annoyed	Somewhat more easily annoyed	Much more easily annoyed	4

PART 3: Please answer the following questions that pertain to the varying sound levels in your home using the following scale:

1 = Very much like our home
2 = Somewhat like our home
3 = A little bit like our home
4 = Not at all like our home

1) A sound device is always on in our home (T.V., radio, iPad, etc)	1	2	3	4
2) We never have more than one sound device on at a time in our home	1	2	3	4
3) We always have guests in our home	1	2	3	4
4) We never hear sirens from emergency vehicles passing by outside	1	2	3	4
5) We never raise our voice to talk to each other when we're in different rooms	1	2	3	4
6) We always have our mobile phones set to silent while in our home	1	2	3	4
7) People are often leaving and entering our home	1	2	3	4
8) We can hear traffic noise from vehicles passing by outside	1	2	3	4
9) The street that we live on does not have any traffic	1	2	3	4
10) The rooms used most often when in our home are left with their doors open	1	2	3	4
11) The doors in our home open and close very quietly	1	2	3	4
12) We often receive and make phone calls while at home	1	2	3	4

13)	We never hear planes or helicopters passing over us	1	2	3	4
14)	We have a pet that makes a lot of noise	1	2	3	4
15)	We vacuum our house often	1	2	3	4
16)	We can often hear people outside while we are in our home	1	2	3	4
17)	The children's toys/games in our home don't make any noise	1	2	3	4
18)	We can never hear sounds from our kitchen when in other rooms in our home	1	2	3	4
19)	We are often preparing things in our kitchen	1	2	3	4
20)	We never leave our windows or doors to the outside open while in our home	1	2	3	4
21)	When the washing machine is running, we can always here it from other rooms in our home	1	2	3	4
22)	We can't hear any of the clocks in our home	1	2	3	4
23)	We can hear our neighbours in their home while we're inside of our home	1	2	3	4
24)	Someone in our home has a hobby that makes a lot of noise, and we can always hear them when they are working on it (example: playing an instrument, woodworking, video gaming)	1	2	3	4
25)	We can't hear running water (example: shower, toilet, sink) when in other rooms in our home	1	2	3	4

Please list any other causes of loud sounds that you can hear in your home that we may have missed in the above questions:

____
____
____
____
____

Appendix B

SPSS MANCOVA script with post-hoc analyses using adjusted means and Bonferroni corrections

```
GLM Flanker_IES Flanker_ΔRT Flanker_Δcongruency WCST_PerseverativeErrors WCST_NonPersevErrors
   WCST_SetFailure WCST_TotalErrors BDS_FinalScore BDS_ProportionCorrect BY NoiseCondition
   HomeNoiseGroup AgeGroup WITH Country
  /METHOD=SSTYPE(3)
  /INTERCEPT=INCLUDE
  /EMMEANS=TABLES(NoiseCondition) WITH(Country=MEAN) COMPARE ADJ(BONFERRONI)
  /EMMEANS=TABLES(HomeNoiseGroup) WITH(Country=MEAN) COMPARE ADJ(BONFERRONI)
  /EMMEANS=TABLES(AgeGroup) WITH(Country=MEAN) COMPARE ADJ(BONFERRONI)
  /EMMEANS=TABLES(NoiseCondition*HomeNoiseGroup) WITH(Country=MEAN)
  /EMMEANS=TABLES(NoiseCondition*AgeGroup) WITH(Country=MEAN) compare (AgeGroup) ADJ(BONFERRONI)
  /EMMEANS=TABLES(AgeGroup*NoiseCondition) WITH(Country=MEAN) compare (NoiseCondition) ADJ(BONFERRONI)
  /EMMEANS=TABLES(HomeNoiseGroup*AgeGroup) WITH(Country=MEAN) compare (AgeGroup) ADJ(BONFERRONI)
  /EMMEANS=TABLES(AgeGroup*HomeNoiseGroup) WITH(Country=MEAN) compare (HomeNoiseGroup) ADJ(BONFERRONI)
  /EMMEANS=TABLES(NoiseCondition*HomeNoiseGroup*AgeGroup) WITH(Country=MEAN)
  /PRINT=DESCRIPTIVE ETASQ
  /CRITERIA=ALPHA(.05)
  /DESIGN=Country NoiseCondition HomeNoiseGroup AgeGroup NoiseCondition*HomeNoiseGroup
   NoiseCondition*AgeGroup HomeNoiseGroup*AgeGroup NoiseCondition*HomeNoiseGroup*AgeGroup.
```
